# Factors associated with less-than-full-time working in medical practice: results of surveys of five cohorts of UK doctors, 10 years after graduation

**DOI:** 10.1186/s12960-016-0162-3

**Published:** 2016-10-13

**Authors:** Shelly Lachish, Elena Svirko, Michael J. Goldacre, Trevor Lambert

**Affiliations:** Nuffield Department of Population Health, UK Medical Careers Research Group, Unit of Health-Care Epidemiology, University of Oxford, Old Road Campus, Oxford, OX3 7LF United Kingdom

**Keywords:** Doctors’ working patterns, Part-time, Less-than-full-time, Children, Family, Seniority, Specialty, Healthcare workforce planning, Gender differences

## Abstract

**Background:**

The greater participation of women in medicine in recent years, and recent trends showing that doctors of both sexes work fewer hours than in the past, present challenges for medical workforce planning. In this study, we provide a detailed analysis of the characteristics of doctors who choose to work less-than-full-time (LTFT). We aimed to determine the influence of these characteristics on the probability of working LTFT.

**Methods:**

We used data on working patterns obtained from long-term surveys of 10,866 UK-trained doctors. We analysed working patterns at 10 years post-graduation for doctors of five graduating cohorts, 1993, 1996, 1999, 2000 and 2002 (i.e. in the years 2003, 2006, 2009, 2010 and 2012, respectively). We used multivariable binary logistic regression models to examine the influence of a number of personal and professional characteristics on the likelihood of working LTFT in male and female doctors.

**Results:**

Across all cohorts, 42 % of women and 7 % of men worked LTFT. For female doctors, having children significantly increased the likelihood of working LTFT, with greater effects observed for greater numbers of children and for female doctors in non-primary care specialties (non-GPs). While >40 % of female GPs with children worked LTFT, only 10 % of female surgeons with children did so. Conversely, the presence of children had no effect on male working patterns. Living with a partner increased the odds of LTFT working in women doctors, but decreased the odds of LTFT working in men (independently of children). Women without children were no more likely to work LTFT than were men (with or without children). For both women and men, the highest rates of LTFT working were observed among GPs (~10 and 6 times greater than non-GPs, respectively), and among those not in training or senior positions.

**Conclusions:**

Family circumstances (children and partner status) affect the working patterns of women and men differently, but both sexes respond similarly to the constraints of their clinical specialty and seniority. Thus, although women doctors comprise the bulk of LTFT workers, gender is just one of several determinants of doctors’ working patterns, and wanting to work LTFT is evidently not solely an issue for working mothers.

**Electronic supplementary material:**

The online version of this article (doi:10.1186/s12960-016-0162-3) contains supplementary material, which is available to authorized users.

## Background

Medical workforce planning is a complex but essential process in the provision of high-quality health services to meet the needs of the population. Along with the number of graduates recruited and the number of doctors retiring or leaving the profession, the level of full-time working is a key determinant of medical workforce supply. Workforce shortages arise not just from an undersupply of doctors to training or consultant posts but also from an increase in the numbers working less-than-full-time (LTFT) hours.

The persistence of traditional gender roles in society, in which women undertake the bulk of family caring responsibilities, means that female doctors typically work fewer hours than their male counterparts: this pattern transcends medicine [1–3]. Such gendered working patterns are important considerations for medical workforce planners, particularly given women’s increasing representation in the medical workforce [2]. Recent studies, however, have shown that doctors of both sexes are working fewer hours than in the past [4–6], with male doctors showing the greater rate of decline in working hours [7, 8]. In the UK, requests to enter LTFT training have increased, facilitated by recent legislation granting all employees the right to request flexible and LTFT working hours (not just parents) [4, 9]. Authors of some studies have indicated that this move to reduced working hours for doctors represents a cultural shift in valuing time over money and prioritising work-life balance above career progression [7, 10].

Major changes in doctors’ working patterns, including the increased use of LTFT work, are a challenge to planning of the long-term provision of effective public health services [11, 12]. Planning for a sustainable future medical workforce requires detailed knowledge of the characteristics of doctors who choose to work less-than-full-time. Apart from gender, studies have identified several relevant characteristics including family circumstances, marital status and professional specialism [10, 13]. However, most studies have examined sections of the general working population rather than doctors specifically, and many have examined factors in isolation rather than jointly.

Our aim in this study was to determine the relative influence of a number of personal and professional characteristics of doctors on the probability of working LTFT. For this, we used data on working patterns obtained from long-term surveys of more than 11,000 UK-trained doctors.

## Methods

### Establishing employment histories and working patterns

Since 1975, the UK Medical Careers Research Group has followed the careers of UK doctors by conducting postal and, more recently, web surveys at regular intervals after graduation [14]. The starting point for surveys is the cohort of qualifiers from all UK medical schools in selected years (contact details are supplied by the UK General Medical Council). Cohorts are surveyed towards the end of their first post-graduate year, at 3, 5, 7, 11 years post-graduation, and at longer intervals after that.

Our multipurpose questionnaires ask doctors about topics related to their career and work experiences. Doctors are also asked to provide details of their current and past employment, including the duration and dates of positions, the medical specialty, grade and location of the positions, and whether they were undertaken on a full-time or less-than-full-time basis. For our analyses, we used the position held by each doctor on 30 September of each year to construct an annual employment record. For the small number of respondents who reported multiple concurrent jobs, we included the job with the highest priority based on a ‘scoring system’ similar to that used by the Department of Health in England (for example, permanent posts were prioritised ahead of locum appointments, posts in medicine were prioritised ahead of posts outside medicine). As doctors provided information on the start and end dates of their jobs, we can populate doctors’ annual employment records for the years between our surveys.

We analysed working patterns at 10 years post-graduation (when most doctors are well advanced in a specialty) for doctors of five graduating cohorts, 1993, 1996, 1999, 2000 and 2002. Hence, we analysed the work patterns in the years 2003, 2006, 2009, 2010 and 2012, respectively. Based on information provided by respondents, we had information on employment at 10 years post-graduation for 53 % of the 20,616 doctors who graduated in these five years (*N* = 10,866; see Table 1).

**Table 1:** Numbers of doctors with known career destinations and working patterns 10 years post-graduation

	Cohort (year of graduation)					
	1993^a^	1996	1999	2000	2002^b^	Total
Doctors in the graduating cohort	3671	3868	4213	4428	4436	20,616
Doctors with known career destination	2690	1978	2226	2244	2048	11,186
Doctors with known working patterns	2607	1886	2192	2191	1990	10,866
% of graduating cohort	71.0	48.8	52.0	49.5	44.9	52.7

For those whose employment record at 10 years post-graduation was unknown, we used information on employment at either 9 years post-graduation (*N* = 197 doctors) or at 11 years post-graduation (*N* = 94 doctors)

^a^The 1993 cohort has been surveyed many more times than subsequent cohorts enabling us to hold more extensive information about their careers

^b^The 2002 cohort has been affected by changes to GMC rules about their permissions for us to contact doctors

### Establishing personal and professional characteristics

In this paper, we have used the term LTFT to denote less-than-full-time training and working, rather than the term ‘part-time’. In medical training in the UK, the definition and recognition of LTFT training arises in European Union law (directive EC directive 93/16/EEC, see http://www.aic.lv/ace/ace_disk/Recognition/dir_prof/SECTORAL/93_16Doct.pdf) and is characterised by being at least 50 % of full-time hours. The term ‘part-time’ could describe something less formal and, in particular, less than 50 %.

To investigate how LTFT working varied across clinical specialties, we aggregated the specialties indicated by respondents in their employment histories into four broad specialty groups: general practice (GP), hospital medical specialties (Hosp), surgical specialties (Surg), and other clinical specialties (Other; see Additional file 1: Table S1).

To assess the influence of seniority on the probability of working LTFT, we categorised the job grades indicated by respondents in their employment histories as: trainee (post-graduate training grades, including these UK National Health Service (NHS) designations: core trainees, specialist trainees, registrars, house officers, assistants, fellows, tutors), senior (including consultants, principals in general practice, directors, professors), and career (all other grades, notably doctors who had finished specialty training but whose job did not involve the full responsibilities of consultants or principals).

We also asked doctors to answer the following six questions: (i) Did you obtain any qualifications before entering medical school? or (ii) Did you obtain any non-clinical qualifications during medical school?; (iii) Where did you live at the time of your application for medical school?; (iv) Do you live with a spouse or partner?; (v) How many children under 16 reside in your household?; (vi) Are there any dependent adults in your household whose needs affect your ability to pursue your chosen career?

Based on respondents’ answers to these questions, we defined the following six factors that we hypothesised may influence the probability that doctors work LTFT: (1) graduate entrant status (binary variable; yes/no), indicating whether the doctor had a degree on entering medical school, and serving as a proxy for age as we did not have accurate age information for many doctors; (2) intercalated degree status (binary variable, yes/no), indicating whether the doctor obtained a research degree during their undergraduate years; (3) family home location at time of entering medical school (binary variable, UK/non-UK); (4) partner status (binary variable, living with spouse or partner/not living with spouse or partner); (5) number of children (ordinal variable with three categories, none, 1, ≥2); and (6) dependent adults in the household (binary variable; yes/no). For factors that could change value over time (variables 4, 5, 6 above), we used information given by respondents in the surveys conducted closest to, but following, the 10 years post-graduation time point (for the 1993, 1996, 1999, 2000 and 2002 cohorts, we used data from surveys conducted in 2004, 2007, 2012, 2012 and 2013, respectively).

### Statistical analyses

As a preliminary inspection of the data showed substantially higher rates of LTFT working in female doctors than in males (Table 1), we fitted regression models to female and male data separately. This facilitated both model fitting and parameter interpretation and avoided the need to include higher-order interactions between variables in multivariable models. We used chi-square tests to determine the strength of association between single factors (cohort, specialty, job grade, graduate status, intercalated degree status, family home location, partner status, number of children, and presence of dependent adults) and the probability of working LTFT 10 years post-graduation. Then, to determine the independent influence of the different factors taking account of other factors, and to assess potential interactions among them, we fitted multivariable logistic regression models to our data. Our starting multivariable models included all factors that were associated with LTFT working in univariable testing (*P* < 0.10) and relevant two-way interactions between factors where we hypothesised such interactions would occur (see Appendix for details). Starting models were optimised by backward stepwise elimination of nonsignificant terms, beginning with higher-order interactions using Wald statistics to assess statistical significance of model covariates (*P* < 0.05) and arrive at the minimum adequate models (see Appendix for details). We present odds ratios (with 95 % CI) for the effect of each parameter on the probability of working LTFT in female and male doctors.

## Results

### Doctors who were working, or not working, in the NHS

We confine our main analyses, following this short section, to doctors working in the UK National Health Service (NHS; including those with honorary NHS contracts who were predominantly employed in clinical academic posts), because they constituted the vast majority of our dataset and are homogeneous in respect of NHS working conditions (91 %; 9868/10,866; Table 1). The 998 doctors working outside the NHS comprised 624 (5.7 %) who were working in medicine outside the UK and 291 (2.7 %) who were working in non-NHS UK medicine, with 54 (0.5 %) in non-medical employment and 28 (0.3 %) not in employment. Among those in medicine abroad, 2.4 % (213/547) of men and 19.5 % (88/451) of women were working LTFT. Among those in non-NHS UK medicine, 2.2 % (4/181) of men and 22.7 % (25/110) of women were working LTFT. Small counts do not permit further subgroup analysis of non-NHS doctors.

### Doctors working in the NHS

- Percentage of doctors working LTFT
  Across all five cohorts, 42.1 % (95 % CI 40.8–43.4 %) of women and 6.7 % (5.9–7.4 %) of men were working LTFT. In each cohort, LTFT working was far more common among female doctors than among male doctors (Table 2). The proportion of female doctors who worked LTFT at 10 years post-graduation was greater in the two earlier cohorts (1993, 1996) than in the three later cohorts (Table 1).

**Table 2:** Percentages of doctors in different categories working less-than-full-time (LTFT), 10 years post-graduation

	Females		Males	
Categories	% LTFT	Number	% LTFT	Number
Cohort				
1993	50.3	1164	6.4	1161
1996	51.8	926	10.2	804
1999	36.6	1077	5.3	905
2000	39.3	1089	4.8	912
2002	33.5	1112	7.1	718
Chi-square	*χ* ^2^_4_ = 119.5, *P* < 0.001		*χ* ^2^_4_ = 24.2, *P* < 0.001	
Specialty group				
General practice	61.7	2360	15.7	1245
Hospital specialties	28.3	735	3.0	804
Surgical specialties	19.0	605	2.0	1148
Other specialties	28.7	1615	4.0	1286
Chi-square	*χ* ^2^_3_ = 679.1, *P* < 0.001		*χ* ^2^_3_ = 239.5, *P* < 0.001	
Job grade				
Senior	41.6	1580	8.0	1591
Career	62.9	1484	20.0	515
Trainee	29.4	2188	2.6	2270
Chi-square	*χ* ^2^_2_ = 406.9, *P* < 0.001		*χ* ^2^_2_ = 215.5, *P* < 0.001	
Family home location				
UK	42.8	4941	6.8	4028
Non-UK	34.2	158	6.1	131
Chi-square	*χ* ^2^_2_ = 4.4, *P* = 0.04		*χ* ^2^_2_ = 0.02, *P =* 0.90	
Intercalated degree				
Yes	41.9	1863	5.6	1814
No	40.9	2578	7.5	1865
Chi-square	*χ* ^2^_2_ = 0.3, *P =* 0.60		*χ* ^2^_2_ = 5.0, *P =* 0.03	
Graduate status				
Yes	44.8	362	11.5	358
No	42.5	4741	6.3	3831
Chi-square	*χ* ^2^_2_ = 0.6, *P =* 0.43		*χ* ^2^_2_ = 13.3, *P =* <0.001	
Living with spouse				
Yes	46.4	4420	6.1	3876
No	18.9	813	10.2	482
Chi-square	*χ* ^2^_2_ = 210.6, *P =* <0.001		*χ* ^2^_2_ = 11.0, *P =* 0.001	
Children				
None	18.0	1635	7.6	1275
One	38.7	1090	6.7	871
Two or more	61.1	2413	5.9	2155
Chi-square	*χ* ^2^_2_ = 750.5, *P =* <0.001		*χ* ^2^_2_ = 3.6, *P =* 0.16	
Dependent adults				
Yes	40.2	423	7.9	304
No	42.4	4582	6.6	3856
Chi-square	*χ* ^2^_2_ = 0.7, *P =* 0.40		*χ* ^2^_2_ = 0.6, *P =* 0.44	

**Fig. 1:**
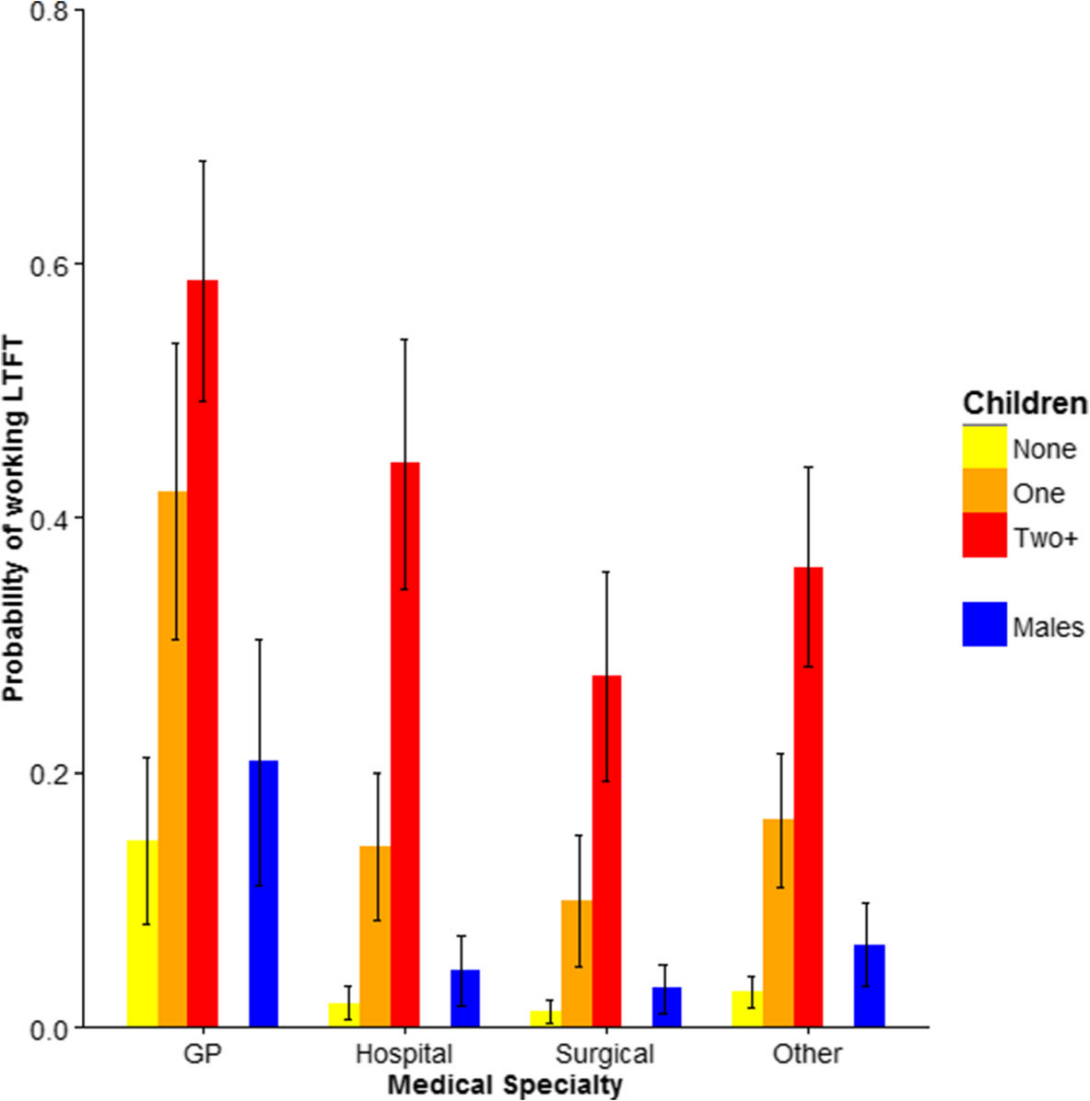
Effect of children on the probability of female doctors in different medical specialties working LTFT. Also shown are the probabilities of working LTFT for male doctors in those specialties (in *blue*). The plotted predicted probabilities were obtained from multivariable models parameterised for the 2002 cohort with the other covariates held at their reference value (i.e. not living with a spouse/trainee job grade/non-graduate)

Totals are the numbers of female and male doctors in each category and may not sum to equivalent values across all categories due to missing data
- Variation in the probability of working LTFT by single factors
  Results of univariable analyses revealed similarities and differences between female and male doctors in the characteristics associated with the probability of working LTFT (percentages of doctors working LTFT in the different categories are given in Table 2). For both sexes, the probability of working LTFT varied significantly among the five cohorts, among the broad specialty groups, with job grade, and between doctors with and without partners (Table 2). The number of children a doctor had, and to a lesser degree family home location, was associated with the LTFT for women, but not for men (Table 2). Female doctors who worked LTFT had almost twice as many children (mean = 1.7, SE = 0.02) as females who worked FT (mean = 0.94 SE = 0.02; *P* < 0.001), unlike their male colleagues (FT = 1.34 SE = 0.07; LTFT = 1.38, SE = 0.02; *P* = 0.51). Graduate status, and to a lesser degree intercalated degree status, was associated with the probability of working LTFT only for men; graduates and those with intercalated degrees were more likely to work LTFT (Table 2).
- Multivariable analysis of the probability of working LTFT
  Multivariable models confirmed that for both men and women, the highest rates of LTFT working were observed among GPs (Fig. 1). The odds of working LTFT were on average 10 times higher for female GPs than for female non-GPs, and on average 6 times higher for male GPs than for male non-GPs (Table 3; Fig. 1). There was much less variation by gender in the probability of working LTFT among the three non-primary care specialty groups (Table 3; Fig. 1).

**Table 3:** Multivariable effects of personal and professional characteristics on probability that doctors work LTFT, 10-years post-graduation

	Females^b^		Males^c^	
Model terms^a^	Wald *χ* ^2^	Odds ratio (95 % CI)	Wald *χ* ^2^	Odds ratio (95 % CI)
Cohort [2002]	*χ* ^2^ = 172, df = 4, *P* < 0.001	1.00	*χ* ^2^ = 18.9, df = 4, *P* < 0.001	1.00
1993		2.53 (2.05–3.12)		0.83 (0.55–1.28)
1996		2.71 (2.17–3.39)		1.31 (0.86–2.01)
1999		0.93 (0.75–1.14)		0.70 (0.43–1.12)
2000		1.17 (0.95–1.44)		0.54 (0.33–0.88)
Specialty [GP]	*χ* ^2^ = 103.0, df = 3, *P* < 0.001	1.00	*χ* ^2^ = 89.2, df = 3, *P* < 0.001	1.00
Hospital		0.11 (0.06–0.21)		0.18 (0.10–0.30)
Surgical		0.07 (0.03–0.15)		0.12 (0.07–0.20)
Other		0.16 (0.10–0.25)		0.26 (0.18–0.39)
Job Grade [Trainee]	*χ* ^2^ = 37.5, df = 2, *P* < 0.001	1.00	*χ* ^2^ = 60.6, df = 2, *P* < 0.001	1.00
Career		3.53 (2.14–5.86)		3.51 (2.30–5.39)
Senior		1.50 (0.91–2.49)		1.11 (0.73–1.69)
Living with Spouse [No]	*χ* ^2^ = 4.10, df = 1, *P* = 0.04	1.00	*χ* ^2^ = 16.3, df = 1, *P* < 0.001	1.00
Yes		1.31 (1.01–1.71)		0.46 (0.32–0.68)
Graduate Status [No]	NA	NA	*χ* ^2^ = 11.1, df = 1, *P* < 0.001	1.00
Yes		NA		1.96 (1.30–2.89)
Children [None]	*χ* ^2^ = 52.6, df = 2, *P* < 0.001	1.00	NA	NA
One		4.23 (2.24–8.05)		NA
Two or more		8.26 (4.66–14.74)		NA
Specialty*Children [GP*None]	*χ* ^2^ = 28.6, df = 6, *P* < 0.001	1.00	NA	NA
Hospital*1		1.99 (0.92–4.50)		NA
Surgical*1		2.06 (0.80–5.71)		NA
Other*1		1.62 (0.91–2.92)		NA
Hospital*2+		4.91 (2.46–10.41)		NA
Surgical*2+		3.66 (1.60–9.30)		NA
Other*2+		2.43 (1.46–4.09)		NA
JobGrade*Children [Trainee*None]	*χ* ^2^ = 11.0, df = 4, *P* = 0.03	1.00	NA	
Career*1		0.43 (0.23–0.83)		NA
Senior*1		0.49 (0.26–0.93)		NA
Career*2+		0.41 (0.23–0.73)		NA
Senior*2+		0.39 (0.22–0.70)		NA

Results of separate multivariable logistic regression models performed for female and male doctors: terms with ‘NA’ in cells were not included in the final model for that sex

^a^The reference category of each model term and interaction is given in square brackets

^b^The final model for female doctors was [~Cohort+JobGrade+Spouse+Specialty*Children+JobGrade*Children]

^c^The final model for male doctors was [~Cohort+JobGrade+Spouse+Specialty+GradStatus]

Models also showed that the presence of children in the family home increased the probability that female doctors worked LTFT and that the extent to which children affected LTFT working differed by specialty group (Table 3). Compared to female GPs with no children, female GPs with one child were on average four times as likely to be working LTFT, while those with two or more children were on average eight times as likely to be working LTFT (Table 3; Fig. 1). For females in the non-primary care specialties, the presence of children increased the likelihood of working LTFT over those without children to a far higher degree (Table 3). Nonetheless, predicted rates of LTFT working with children were still lower for females in non-primary care than for female GPs (Fig. 1). For example, while >40 % of female GPs with one child worked LTFT, only 10 % of female surgeons with one child did so (Fig. 1). Importantly, the working patterns of female doctors with no children did not differ significantly from those of male doctors (with or without children) in any of the specialty groups (Fig. 1).

**Fig. 2:**
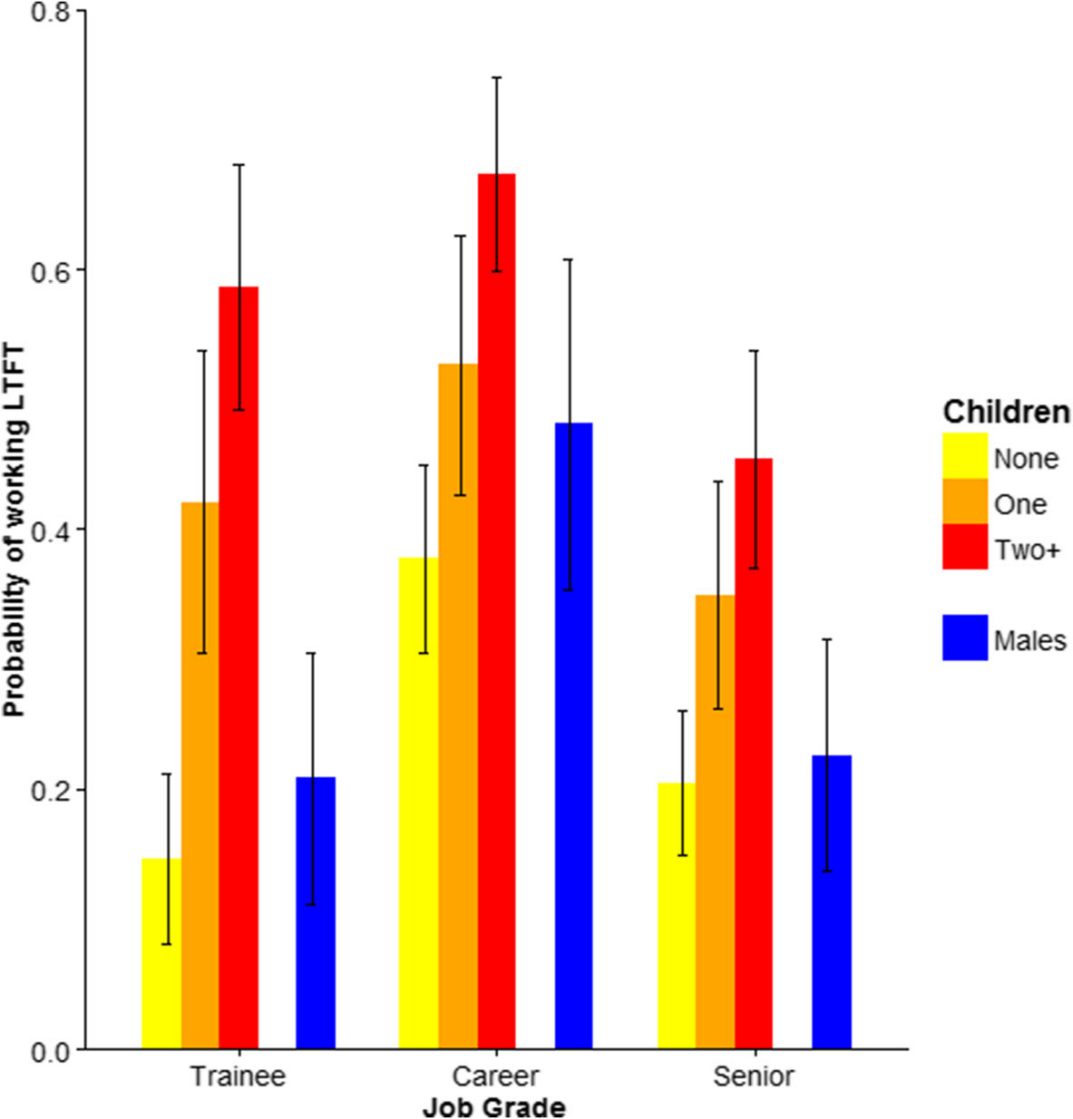
Effect of children on the probability of female doctors in different job grades working LTFT. Also shown are the probabilities of working LTFT for male doctors in those job grades (Trainee, Career, Senior). Predicted probabilities obtained from multivariable models parameterised for the 2002 cohort. Other covariates in models were held at their reference values: GP/not living with spouse/non-graduates)

Among women, the effect of having children on the likelihood of working LTFT varied marginally according to job grade (*P* = 0.03; Table 3). Both male and female doctors in ‘career’ grade jobs were more likely to work LTFT than were doctors in trainee positions or in senior positions (Table 3, Fig. 2). Children, however, increased the likelihood of working LTFT to a greater degree for female trainees than for females in higher-level positions (Table 3, Fig. 2). While women were more likely to be in career grade positions than men (28 vs 11 %), 50 % of doctors in trainee and senior positions were women. There was no evidence of an interaction between job grade and clinical specialty for either sex (see Appendix).

The working patterns of both sexes were affected by the presence of a partner, but in opposite ways. Living with a partner increased the odds of LTFT working in females by 31 %, but decreased the odds of LTFT working in males by 54 % (Table 3). The effect of partner status on working LTFT did not differ by clinical specialty, family home location (for female doctors) or graduate status (for male doctors; see Appendix). However, male doctors who were graduate entrants to medical school, and thus on average older than non-graduate entrants, were twice as likely to work LTFT as were those who had not undertaken a prior degree (odds ratio = 2.0, CI 1.3–2.9; Table 3).

## Discussion

In this study of practising doctors, almost half of the women (42 %) but few men (7 %) worked LTFT. We showed that a substantial proportion of this gender variation in doctors’ working patterns could be attributed to the presence of children and a spouse in the family home. Moreover, our study also showed that while family circumstances (children and partner status) affect male and female doctors’ working patterns in contrasting ways, professional circumstances (specialty and seniority) influence working patterns in similar ways for both sexes.

Child-rearing responsibilities strongly influenced the probability that female doctors worked LTFT, with substantially greater effects associated with greater numbers of children, but had no effect on the likelihood that male doctors worked LTFT. Indeed, 88 % of the female doctors who worked LTFT in this study had children compared with just 65 % of the male doctors who worked LTFT. Living with a spouse increased the likelihood that female doctors worked LTFT (independently of whether they had children), but decreased the likelihood that male doctors worked LTFT. These results support previous studies showing that parenthood and marriage decrease female doctors’ working hours, but increase or have a negligible effect on men’s working hours [7, 10, 12, 13, 15]. Hence, our work provides further evidence that the persisting female-male difference in LTFT is probably due to persisting unequal division of domestic responsibilities: women in domestic partnerships or with children spend less time at work than men with similar responsibilities [7, 10, 16, 17]. Accordingly, one may surmise, though we do not have direct evidence to support the conjecture, that men, more than women, work LTFT for reasons other than family commitments. Importantly, we showed that female doctors with no children were no more likely to work LTFT than were male doctors, with or without children. This strengthens the notion that female doctors’ working patterns (FT vs LTFT) are largely driven by family caring commitments [18]. This is an essential consideration for workforce planners seeking novel training and employment strategies to accommodate increasing numbers of women within medicine, particularly as studies show that female doctors with older children work as many hours as their male colleagues [19, 20].

A key finding of this study was that the association of LTFT working with having children differed by clinical specialty, with stronger effects for female doctors working in non-primary care than for GPs. Almost all female non-primary care specialists who worked LTFT had children (93 %; compared to 81 % for female GPs). However, women in non-primary care specialties were far less likely to work LTFT than were female GPs with children (e.g. 68 % of female GPs with children worked LTFT compared with just 33 % of female surgeons with children). These are important findings for workforce planners. Previous studies have not directly assessed how the effect of children on doctor’s working patterns varies by specialty. However, several studies have reported higher rates of LTFT working among GPs than among doctors working in other specialties [7, 15, 21]. Here too, we found that GPs were far more likely to work LTFT than other specialty doctors. This was true for both male and female doctors. As we controlled for job grade in our models, the observed differences between specialty groups cannot be explained by variation in the ‘seniority’ of doctors among specialties. Rather, different rates of LTFT working in different specialties occur because specialties differ in demographic composition (e.g. female doctors comprised 60 % of GPs in this study, but only 35 % of surgeons) and in organisational structure (there are greater opportunities for working reduced hours in GP than in most hospital-based specialties). Thus, while personal circumstances may drive doctors’ choices for particular specialties [22], the nature of some specialties may, equally, facilitate particular life choices [6, 17]. Long-term studies examining changes in career preferences, family responsibilities and work outcomes among male and female doctors are needed to disentangle these two possibilities.

Compared to general practice, the provision of LTFT posts in non-primary care specialties is a relatively recent phenomenon. Efforts to provide flexible working and LTFT posts in specialties other than GP in the UK and elsewhere have improved [4]. Nevertheless, our results showing that far fewer female doctors with children work LTFT in the hospital and surgical specialties than in GP and that almost all the LTFT posts in non-primary care are occupied by women with children, suggesting that needs are still not being adequately met in these areas. For female doctors, the possibility of accommodating work and family responsibilities via a LTFT working pattern remains strongly constrained by opportunities to do so in particular specialities [17]. Moreover, as some male GPs and female GPs with no children also work LTFT (Fig. 1), a desire for reduced working patterns is evidently not solely a concern for working mothers. Further research in this field should aim to understand the motivations of doctors without children who work LTFT hours in individual medical specialties: small sample sizes in individual specialties precluded our doing so in this study.

Doctors in trainee and senior positions were less likely to work LTFT than their colleagues in other positions. This contrasts with previous studies showing larger gender differences in LTFT working rates with career progression [23, 24]. Our finding of lower rates of LTFT working for trainees probably reflects the fact that meeting training requirements can be difficult when working LTFT. Medical training is a lengthy process that many doctors do not wish to prolong [25]. In senior positions, LTFT working is challenging, given the greater responsibility, higher workload, lack of specialist expertise cover, and greater administration and management duties involved [24]. After completing specialty training, some doctors may consciously forego senior positions for ‘career’ grade positions that enable them to better balance work and life commitments. In the UK between 2000 and 2010, the number of salaried GPs (which we included in our ‘career’ grade categorisation for analyses) increased tenfold, while the number of principal (senior) GPs declined [26]. This was attributed to a desire among younger GPs for increased working flexibility. Interestingly, our results showed that female doctors with children in trainee positions were more likely to work LTFT than females in higher-level positions. Although the reasons for this are not clear, ensuring that women with family caring responsibilities are accommodated at all stages of their career is an important consideration for future workforce planners [24].

Our analyses suggest that the effect of seniority on working patterns was not driven by age differences among the doctors. Studies examining doctors’ working patterns commonly report that younger and older doctors work fewer hours than middle-aged doctors [7]. Having examined working patterns of doctors at a fixed point in their career, we anticipated less variation in doctors’ ages in our study than occurs in cross-sectional studies covering a wide age range. However, we found that men who were graduate entrants to medical school (and thus on average older than non-graduate entrants) were more likely to work LTFT. Graduate entrants to medical school (and older doctors in general) may be more financially secure if they pursued careers in other fields and have continued interests or responsibilities in areas outside medicine. These factors help explain the increased propensity of male graduate entrants to work LTFT hours, but not why this effect was only present for males. Further work is needed to identify the motivations and career preferences that drive male doctors’ decisions to work LTFT.

Several studies from the UK [9, 19] and elsewhere [5, 27] have reported increasing rates of LTFT working among doctors over past decades. As we examined the working patterns of just five cohorts of medical graduates over a 9-year period (2003 to 2012) at a fixed point in their career (10 years after graduation), our results are not directly comparable to these previous studies. Nevertheless, we observed a decrease in the proportion of female doctors working LTFT in the three later cohorts (1999, 2000 and 2002), with no change in males. Examination of our data do not suggest this change was driven by changes among females in one particular specialty group, though small sample sizes precluded statistical testing of this suggestion. Moreover, this change did not appear to be driven by later cohorts having fewer children or delaying starting a family (females in the five cohorts did not differ in the average number of children they had or the average number of years they had been mothers). An alternative possibility is that the implementation of the European Working Time Directive in 2009, which mandated the reduction of working hours for doctors, and the increased options to work ‘flexibly’ in recent years (e.g. longer hours over fewer days), could have enabled some women with caring responsibilities to balance work-life commitments while remaining essentially ‘full-time’ [28]. Verification of this suggestion and a more thorough exploration of trends in the working patterns of female doctors should be a critical component of planning effective alternative working strategies for the workforce. While women constitute a growing proportion of the medical workforce, working doctors of both sexes increasingly express desires to work fewer hours and for working circumstances that allow time for non-work-related pursuits [9, 29]. This changing face of the medical workforce is a challenge for future effective workforce planning that is facilitated by a greater understanding of the drivers of doctors’ decisions regarding their working patterns.

This study investigated working patterns in a very large number of doctors from across the UK over a 9-year period using information on working patterns obtained independently of any organisation that employs, trains or influences doctors’ careers. Nevertheless, there are limitations to our study. First, we evaluated working patterns in terms of a full-time versus less-than-full-time dichotomy as survey respondents did not specify their weekly hours worked. Examining correlates of hours worked may provide a more nuanced understanding of the drivers of doctors’ working patterns. Second, there may be additional reasons to those we examined influencing doctors’ working patterns. Doctors with concurrent managerial or academic roles, those with health problems or other responsibilities (e.g. sporting or community roles) may only work LTFT in medicine. Third, we assessed working patterns at a fixed point in the doctors’ careers. Understanding how drivers of working patterns change throughout doctors’ careers will require longer-term, continuous monitoring of the employment status, personal and professional characteristics of large numbers of doctors (the subject of our ongoing research). Finally, data on working patterns were derived from self-reported information given in response to surveys. Confidentiality precludes substantiation of the veracity of respondents’ responses, but respondents gained nothing by submitting erroneous data. We cannot, however, discount the potential that non-responders may over-represent those taking non-typical career options.

## Conclusions

We have shown that while family circumstances affect female and male working patterns differently, both sexes respond similarly to the circumstances of their professional niche. Thus, although women comprise the majority of LTFT workers in medicine, gender is just one of several determinants of doctors’ working patterns. Given the growing percentage of women in the medical workforce, and as doctors who work LTFT report being less stressed and more satisfied at work [30], it is time for public health services to acknowledge that LTFT medical careers are here to stay. At the least this should involve creating LTFT appointments across the breadth of medical specialties, implementing policies that encourage women to return to full-time work, and establishing legitimate career paths that enable doctors of both sexes to train and work LTFT.

## Appendix

### Multivariable modelling methods

On the basis of the results of univariable results presented in Table 2, we constructed multivariable models to explain variation in LTFT working.

For female doctors, our starting model contained the additive effects of cohort, specialty, job grade, family home, partner status and number of children, along with eight interactions between covariates (specialty*job grade, specialty*partner, specialty*children, specialty*family home, children*job grade, children*family home, partner*family home, and partner*children). For male doctors our starting model contained the effect of cohort, specialty, job grade, partner status, graduate status and degree status, along with four interactions (specialty*job grade, specialty*partner status, specialty*graduate status, and graduate status*partner status). Small sample sizes in some category combinations precluded inclusion of higher order interactions.

These full starting models were optimised by backward stepwise elimination of nonsignificant terms, beginning with higher order interactions and using Wald’s statistics to assess statistical significance of the model covariates (*P* < 0.05) and arrive at the minimum adequate models. To verify the minimum models, variables not originally selected were added back one at a time to confirm their effects remained negligible in the presence of potential confounders.

After model simplification, the minimum adequate model for female doctors contained the additive effects of cohort, specialty, job grade, partner status and number of children, with the effect of children varying both by specialty and by job grade (Table 4). The minimum adequate model explaining variation in probability of working LTFT for male doctors contained only the additive effects of cohort, specialty, job grade, partner status and graduate status (Table 5). Adding any of the excluded factors back in to these models did not improve model fit.

**Table 4:** Model simplification by backwards stepwise selection of multivariable binary logistic model to assess effects of personal and professional characteristics on the probability of working less-than-full-time, 10 years post-graduation, for female doctors

Model selection steps	Terms removed	Wald’s test^a^		
		*χ* ^2^	df	*P*
1 (Starting model)	Cohort+Specialty+Children+JobGrade+Spouse+FamilyHome+Specialty*Children+Specialty*Spouse+Specialty*FamilyHome+Specialty*JobGrade+JobGrade*Children+Spouse*Children+Children*FamilyHome+Spouse*FamilyHome			
2	- Specialty*Spouse	0.47	3	0.93
3	- FamilyHome*Children	0.61	2	0.74
4	- FamilyHome*Spouse	0.00	1	0.99
5	- Specialty*FamilyHome	1.60	3	0.65
6	- Specialty*JobGrade	4.30	6	0.63
7	- Spouse*Children	1.70	2	0.44
8	- FamilyHome	0.01	1	0.94
7	- Spouse*Children	1.70	2	0.44
8	- FamilyHome	0.01	1	0.94

^a^Results of Wald’s test for removal of specified model term from the model

**Table 5:** Model simplification by backwards stepwise selection of multivariable binary logistic model to assess effects of personal and professional characteristics on the probability of working less-than-full-time, 10 years post-graduation, for male doctors selection

Model selection steps	Terms/terms removed	Wald’s test^a^		
		*χ* ^2^	df	*P*
1 (Full model)	Cohort+Specialty+JobGrade+Spouse+Intercalated Degree+Graduate+Specialty*JobGrade+Specialty*Graduate+Specialty*Spouse+Graduate*Spouse			
2	- Graduate*Spouse	0.02	1	0.88
3	- Specialty*Graduate	3.00	3	0.39
4	- Specialty*Spouse	3.40	3	0.34
5	- Intercalated Degree	0.07	1	0.79
6	- Specialty*JobGrade	11.5	6	0.07

^a^Results of Wald’s test for removal of specified model term from the model

## Additional file

Additional file 1: Table S1. Broad specialty groups used in analyses and 483 the individual specialties in 484 each group. (DOCX 11 kb)
