# Supplementary material for: Factors associated with less-than-full-time working in medical practice: results of surveys of five cohorts of UK doctors, 10 years after graduation
Source: Hum Resour Health. 2016 Oct 13;14:62. doi: 10.1186/s12960-016-0162-3 (PMC5064899; doi:10.1186/s12960-016-0162-3)
Supplement: Additional file 1: Table S1. — Broad specialty groups used in analyses and 483 the individual specialties in 484 each group. (DOCX 11 kb) [file 12960_2016_162_MOESM1_ESM.docx]

**Additional file 1 Table S1:** Broad specialty groups used in analyses and the individual specialties in each group.

| **Broad Specialty Group** | **Specialties, as specified by the doctors, included in each group** |
| --- | --- |
| General Practice [GP] | GP, including ‘GP hospital based’ and ‘GP with psychiatry’ and GP with other interests. |
| Hospital Medicine [HOSP] | Cardiology, Dermatology, Endocrinology, Gastroenterology, Geriatric Medicine, Infectious Diseases, Nephrology, Neurology, Occupational Health, Rheumatology, Rehabilitation, Tropical Medicine, Vascular Medicine, and including general hospital medicine, Medicine ‘unspecified’ |
| Surgical specialties [SURG] | Academic Surgery, Cardiac surgery, Dental surgery, Emergency Medicine, ENT, General surgery, Neurosurgery, Ophthalmology, Orthopaedics/Trauma, Paediatric surgery, Plastic surgery, Urology, Vascular surgery, Obstetrics and Gynaecology, and including ‘general surgery’ and ‘surgery unspecified’ |
| Other Medical specialties [OTHER] | Anaesthetics, Academic Work, Clinical Oncology, Community Health, HM Forces, Medicine in Developing Countries, Paediatrics, Pathology, Psychiatry, Public health, Radiology, and including those who listed two or more specialties. |
